# AcrAB efflux pump impacts on the survival of adherent-invasive *Escherichia coli* strain LF82 inside macrophages

**DOI:** 10.1038/s41598-023-29817-0

**Published:** 2023-02-15

**Authors:** Giulia Fanelli, Martina Pasqua, Gianni Prosseda, Milena Grossi, Bianca Colonna

**Affiliations:** Department of Biology and Biotechnology “C. Darwin”, Istituto Pasteur Italia, Sapienza Università di Roma, Rome, Italy

**Keywords:** Bacteriology, Microbial genetics, Pathogens

## Abstract

The tripartite complex AcrAB-TolC is the major RND pump in *Escherichia coli* and other Enterobacteriaceae. It consists of the AcrB transporter, which is embedded in the inner membrane, the AcrA adapter located in the periplasm, and the channel protein TolC responsible for the transport of substrates towards the extracellular environment. Besides conferring resistance to many classes of antibiotics, AcrAB plays a role in the pathogenesis and virulence of several bacterial pathogens. Here we report that the AcrAB pump heavily affects the infection process of the LF82 strain, the prototype of Adherent-Invasive *Escherichia coli* (AIEC) which are highly abundant in the ileal mucosa of Chron disease patients. We found that the deletion of genes encoding AcrA and/or AcrB leads to decreased survival of LF82 within macrophages. Ectopic AcrAB expression in a *acrAB* defective mutant restores the wild type condition. Furthermore, we demonstrate that inhibition of AcrB and replacement of the transporter with an unfunctional AcrB also interfere with bacterial viability inside macrophages. Overall, these data suggest a pivotal role of the AcrAB efflux pump in bacteria-host cell interactions also in AIEC.

## Introduction

AcrAB belongs to the resistance nodulation cell-division (RND) efflux pump (EP) family, which is primarily responsible for resistance to a broad class of antibiotics contributing to both intrinsic and horizontally acquired multidrug resistance (MDR)^1–4^. It represents the major EP in *Escherichia coli*, *Salmonella,* and other Enterobacteriaceae where it confers resistance to a large panel of different compounds including antibiotics, dyes, detergents, bile salts, and host-derived molecules^5–8^. AcrAB-TolC is a tripartite EP composed by an inner membrane transporter, AcrB, an outer membrane channel, TolC and a periplasmic adaptor protein (PAP), AcrA^9^. The latter protein, in addition to establishing the link between AcrB and TolC, transmits the conformational changes of AcrB to TolC, contributing to the opening of the channel^10,11^. This EP exerts its function by exploiting the proton motive force represented by the electrochemical potential of H^+^ across the inner membrane. The energy transduction and substrate transport are spatially separated inside the AcrB transporter^12–14^. AcrB is an asymmetric homotrimer in which any protomer can assume, not necessarily in a synchronous mode, different conformational states identified as loose (L), tight (T) or open (O)^15,16^. It has been demonstrated that the periplasmic loop of AcrB is responsible for the substrate specificity of the entire tripartite complex^17^. AcrB recognizes its specific substrate through a periplasmic access pocket (AP) in the loose state, while in the tight state the substrate binds to a deeper pocket (DP)^15,18^. AcrAB and its homologues are more widespread than other RND systems and their capability to extrude a large number of different substrates makes them the most clinically relevant among RND EPs^3,19,20^.

An increasing number of data highlights the role of several MDR EPs, including AcrAB, in different steps of the bacterial pathogencity process, spanning survival within the host cells, biofilm formation, and toxin extrusion^21–25^. In this context the AcrAB EP has been described to be relevant for the virulence phenotype of several bacterial pathogens. In particular, in *S. enterica* it has been shown that the deletion of *acrB* or *acrA*, as well as the lack of a functional AcrB transporter, impairs epithelial cell invasion and reduces bacterial virulence in in vivo infection models^21,26,27^. In *Enterobacter cloacae* Complex (ECC), among the many RND EPs present in the genome, only AcrAB is involved in bacterial pathogenicity. Indeed, *E. cloacae acrB* or *acr*A defective strains display attenuated virulence in a *Galleria melonella*^28^ and in mouse infection models^29^. Moreover, inactivation of *acrB* leads to decreased virulence in *Klebsiella pneumoniae*, as demonstrated by the low number of defective bacteria recovered from the spleen of infected mice as compared to the wt strain^30^. Furthermore, *Moraxella catarrhalis* mutants defective in single components of the AcrAB-OprM EP show a decreased infection ability of cell monolayers, suggesting that all components are required for the full invasion of human nasopharyngeal cells in vitro^31^. In *Neisseria gonorrhoeae* the MtrCDE RND efflux system, homologous to the AcrAB-TolC, is required for survival of the pathogen in the female genital tract^32^. The role of AcrAB has been also demonstrated in plant pathogens as *Erwinia amylovora* where AcrAB is an efflux complex required for virulence^33^.

Adherent-Invasive *Escherichia coli* (AIEC) is a group of pathogenic *E. coli* isolated at high frequency in patients suffering of Chron’s disease (CD), a severe intestinal inflammatory disease^34^. Several molecular and cellular studies support a role of AIEC in CD by promoting inflammation via stimulation of the immune system^35–37^. The AIEC phenotype relies on the ability of the bacteria to adhere and invade intestinal epithelial cells and to survive and replicate extensively inside macrophages without inducing cell death^38–40^. While many reports stress the relevance of AcrAB in the virulence of several bacterial pathogens, no data are currently available concerning the possible involvement of this EP in the AIEC pathogenicity program. Therefore, in the present study, we have investigated on the role of the AcrAB EP during the infection process of LF82, the prototype strain of AIEC^34^*.* We have analysed the intracellular viability of LF82 derivatives lacking either the entire *acrAB* operon or the single *acrA* and *acrB* genes. The results from infections of Caco-2 intestinal epithelial cells and of THP-1-derived macrophages suggest that the loss of AcrAB components greatly affects bacterial survival in the macrophage environment, while viability in epithelial cells is only very marginally touched. The requirement of AcrB transporter activity for intramacrophage survival was confirmed by loss of function approaches using either an EP inhibitor or a LF82 strain expressing an unfunctional AcrB form in the infection experiments. Overall, our data indicate that also in AIEC the AcrAB EP is required for the full expression of pathogenicity.

## Results

### The lack of AcrAB affects AIEC LF82 survival inside macrophages

In a previous work^24^ we have analyzed the expression profile of all MDR EPs present in AIEC both under laboratory conditions and during the invasion of epithelial cells and macrophages. The results indicate that the expression of the LF82 *acrA* gene, selected as reporter of the behavior of the *acrAB* operon, is very high both under laboratory conditions as well as in intracellular bacteria. On account of the pivotal role of the AcrAB pump in pathogenic bacteria we asked whether this EP is specifically involved in the infection process of the AIEC LF82 strain. To this end, using the one-step method of gene inactivation^41^, we generated a LF82 derivative carrying a deletion of the entire *acrAB* operon. The extent of the deletion in LF82 *ΔacrAB* was verified by sequencing. In LB medium or in tissue DMEM and RPMI culture media the *ΔacrAB* derivative had a generation time similar to the parental strain (Supplementary Fig. S1). As expected, it showed higher susceptibility to the antibiotics tested (erythromycin, ciprofloxacin, tetracycline) and bile salts (Table 1).

**Table 1:** MICs of AcrAB substrates for LF82 and *acrA*, *acrB* or both and complementary strains.

Strain	MIC of substrate^a^ (µg/ml)			
	ERY	CIP	TET	BS
LF82 (wt)	200	0.25	1	> 1500
LF82 *ΔacrAB*	25	< 0.001	0.25	750
LF82 *ΔacrB*	25	< 0.001	0.25	750
LF82 *∆acrA*	12.5	< 0.001	0.25	750
LF82 *∆acrAB* p*acrAB*	200	0.2	1	> 1500
LF82 *ΔacrB* p*acrB*	200	0.2	1	> 1500
LF82 *ΔacrB* p*acrB*_D408A_	25	0.0015	0.25	750

^a^*ERY* erythromycin, *CIP* ciprofloxacin, *TET* tetracycline, *BS* bile salts.

**Figure 1:**
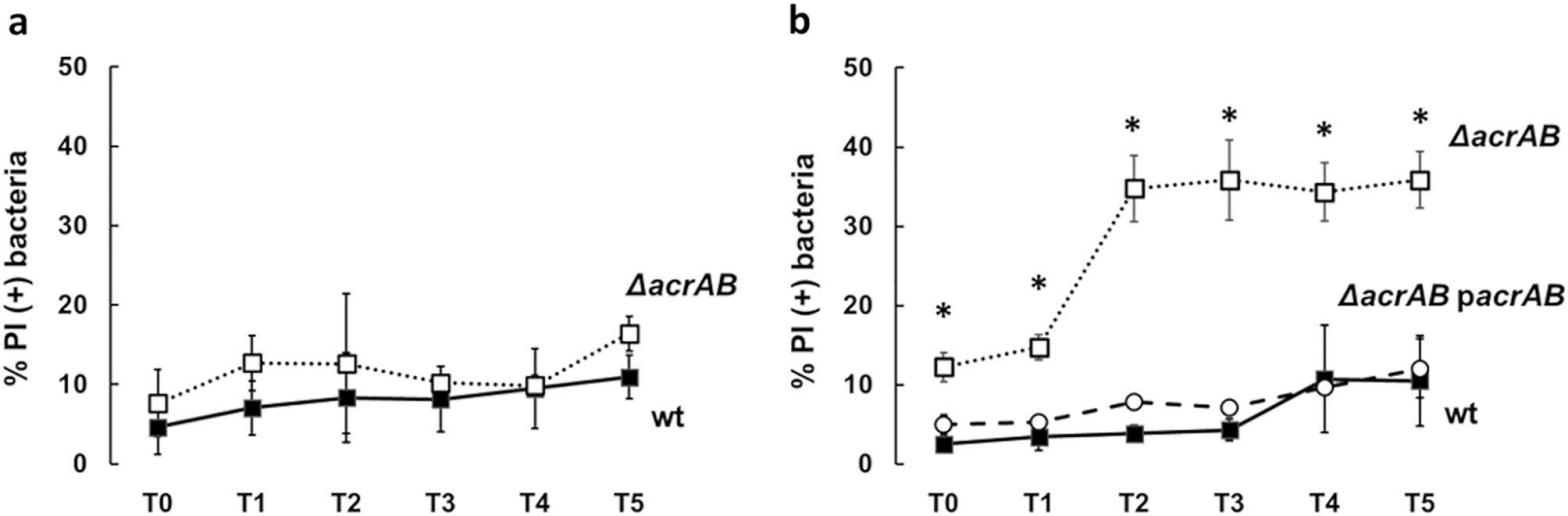
The AcrAB pump affects the intracellular viability of LF82. The viability of intracellular LF82 (black square) and LF82 *ΔacrAB* (open square) bacteria recovered from Caco-2 epithelial cells (**a**) and LF82 (black square**),** LF82 *ΔacrAB* (open square), LF82 *ΔacrAB* p*acrAB* (open circle) recovered from THP-1-derived macrophages (**b**) at 0, 1, 2, 3, 4, and 5 h p.i. (Referred to as T0, T1, T2, T3, T4 and T5, respectively) was assessed by DAPI/PI double staining. The values are expressed as percentage of PI (+) dead bacteria relative to DAPI (+) bacteria. The results are the average of at least three independent experiments. Error bars represent the SD. The statistical significance was determined by a two tailed student’s t test. *p ≤ 0.01.

Infection of Caco-2 epithelial cells and THP-1-derived macrophages were carried out with LF82 or its *acrAB* deletion mutant. To monitor the survival in the intracellular environment, bacteria were recovered at several time points (T0, T1, T2, T3, T4 and T5) post infection (p.i.), and stained with propidium iodide (PI) and DAPI. Subsequently the amount of PI positive bacteria, corresponding to dead cells, was evaluated by fluorescence microscopy. As shown in Fig. 1a most LF82 bacteria, either wt or ∆*acrAB*, infecting Caco-2 cells remain viable throughout the infection period, indicating that lack of AcrAB does not significantly affect the survival of LF82 in the epithelial cell environment. On the contrary, as compared to the LF82 wt strain the absence of AcrAB induces a significant increase of bacteria that die within the THP-1-derived macrophages. The percentage of PI positive LF82 Δ*acrAB* is already very high at early stages of infection (T0 and T1) and reaches dramatically higher values two hours p.i. (T2). Indeed, at this time point the PI positive LF82 Δ*acrAB* cells are more than 30% of the total population. This value remains constant during successive infection time points (Fig. 1b). To confirm the direct contribution of AcrAB to the viability of LF82 in the macrophage environment, we performed infection experiments using the LF82 Δ*acrAB* complemented with p*acrAB*, a recombinant plasmid containing the *acrAB* operon (Supplementary Table S1). The ectopic expression of the *acrAB* operon fully restores the wt phenotype in the Δ*acrAB* derivative. Indeed, we observe a percentage of dead bacteria comparable to that of the wt at each time points of infection (Fig. 1b). These data suggest that in AIEC the efflux pump AcrAB plays a significant role during the pathogenic process. It is reasonable to speculate that AcrAB allows LF82 to resist the acidic environment of macrophages, probably by exporting toxic metabolites and/or signaling molecules necessary to maintain the homeostasis of gene expression involved in different metabolic pathways.

### The transporter AcrB and the adaptor protein AcrA both contribute to the fitness of AIEC LF82 inside macrophages

**Figure 2:**
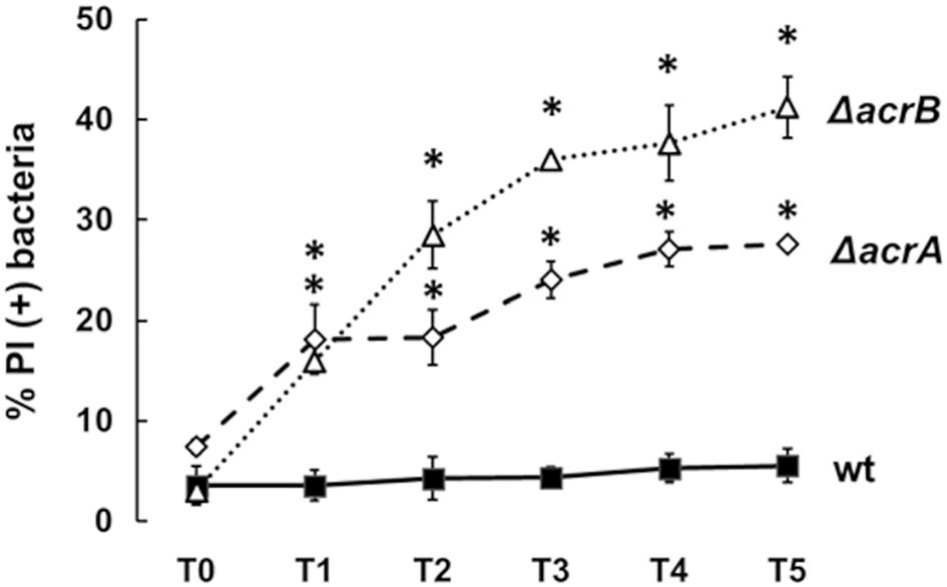
Both the AcrB and AcrA components contribute to survival of LF82 within macrophages. Intracellular LF82 (black square), LF82 *ΔacrB* (open triangle), and LF82 *ΔacrA* (open diamond) bacteria were recovered from THP-1 differentiated into macrophages at 0, 1, 2, 3, 4 and 5 h p.i. (referred to as T0, T1, T2, T3, T4 and T5, respectively) and stained with DAPI/PI. The values are expressed as percentage of PI (+) dead bacteria relative to DAPI (+) bacteria. The results are the average of at least three independent experiments. Error bars represent the SD. The statistical significance was determined by a two tailed student’s t test. *p ≤ 0.01.

It is known that both gene products of the *acrAB* operon contribute to the export of substrates^3^. Indeed, the inner membrane transport protein AcrB is critical for the substrate specificity and the periplasmic adapter protein AcrA is important for connecting AcrB to the TolC channel and transmitting AcrB conformational changes to TolC^3,9^. Based on the results obtained with the LF82 Δ*acrAB* we asked to what extent AcrB and AcrA contribute to the LF82 survival inside macrophages. The entire *acrA* or *acrB* genes were deleted and the growth properties and susceptibility to selected antibiotics of LF82 Δ*acrB* and LF82 Δ*acrA* were assessed (Supplementary Fig. S1 and Table 1). After infecting THP-1-derived macrophages with LF82 Δ*acrB* or LF82 Δ*acrA* the viability of intracellular bacteria compared to the wt was analyzed at different time points p.i. As shown in Fig. 2 throughout the infection period the percentage of LF82 Δ*acrB* PI positive bacteria is higher than in the wt. Indeed, as previously observed in the case of LF82 *ΔacrAB,* the fraction of dead LF82 *ΔacrB* bacteria is much higher than that of the parental strain starting from one hours p.i. (T1) and reaches 40% at five hours p.i. (T5). In addition, Fig. 2 shows that also the lack of AcrA leads to reduced bacterial viability as compared to the wt: the percentage of dead intracellular LF82 *ΔacrA* bacteria is around 20% at T1 and T2 p.i and reaches 27% at five hours p.i. (T5). All together these data reveal an important role of the AcrB transporter in the survival of LF82 within the macrophages, quite possibly as a consequence of a unique transport function for specific host-derived compounds. Moreover, the results suggest that AcrA also contributes, though at a lesser extent, to the fitness of LF82 inside macrophages.

### The AcrB loss of efflux function is responsible for the reduced survival of AIEC LF82 inside macrophages

AcrB is a large abundant transmembrane protein that spans the inner membrane of Gram-negative bacteria. Thus, to ascertain whether the reduced viability of LF82 *ΔacrB* and *ΔacrAB* mutants inside macrophages was due to the absence of this sizeable membrane component or to the loss of its specific efflux function, we inhibited the AcrB activity or used an LF82 strain expressing an unfunctional form of AcrB.

**Figure 3:**
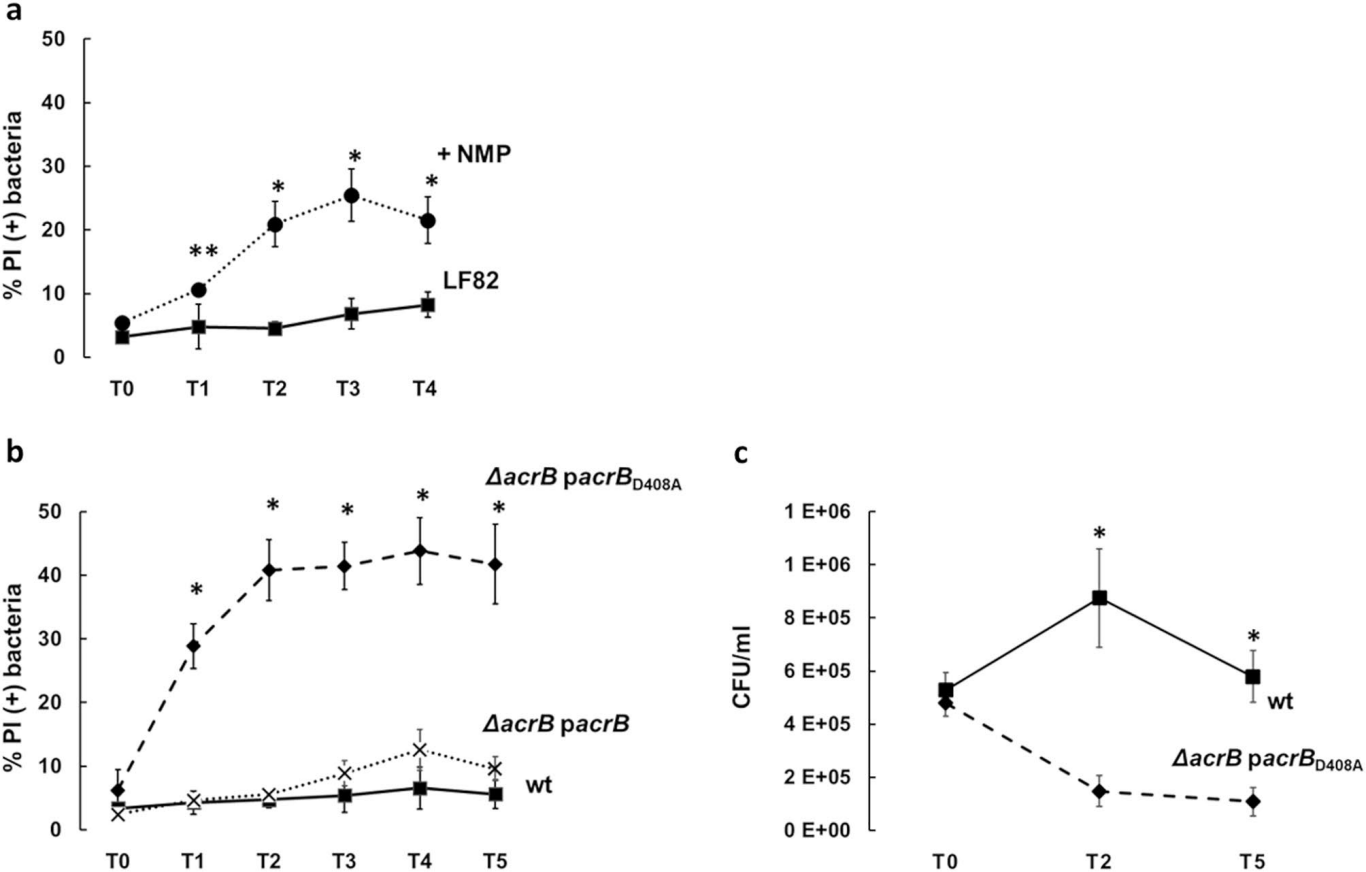
The lack of AcrB function causes loss of LF82 viability inside macrophages. (**a**) Effect of NMP on LF82 survival inside macrophages. THP-1-derived macrophages were infected with LF82 untreated (black square) or treated (black circle) with 100 µg/ml NMP. (**b**) Impact of the presence of the unfunctional AcrB D408A trasporter on LF82 survival within macrophage**s** THP-1 derived macrophages were infected with LF82 (black square) and LF82 ∆*acrB* complemented with either the wild type (x) or the unfunctional (black diamond) AcrB D408A transporter. In (**a**) and (**b**) intracellular bacteria were recovered at the indicated time points and double stained with DAPI/PI. The values are expressed as percentage of PI (+) dead cells relative to DAPI (+) cells. (**c**) Viable counts (CFU/ml) of intracellular LF82 (black square) and LF82 ∆*acrB* complemented with the unfunctional AcrB D408A transporter (black diamond). At the indicated time points, infected THP-1 cells were lysed and dilutions of intracellular bacteria plated on LB agar. The results shown are the average of at least three independent experiments. Error bars represent the SD. The statistical significance was determined by a two tailed student’s t test. *p ≤ 0.01; **p ≤ 0.05.

AcrB function was inhibited by treatment with 1-(1-naphthyl-methyl)-piperazine (NMP), a well characterized efflux pump inhibitor (EPI) in Gram-negative bacteria^42^, used at 100 µg/ml, a concentration known to induce an inhibitory effect on drug efflux in commensal *E. coli*^43^. We verified whether NMP has any influence on the behavior of bacterial and THP-1 single cultures. The inhibitor did not affect LF82 viability nor had toxic effects on THP-1 cells as assessed by measuring LDH (Lactate dehydrogenase) release in the medium (data not shown). To investigate on the phenotype induced by AcrB inhibition we carried out infection experiments of THP-1 cells differentiated into macrophages with the LF82 wt strain in the presence or absence of NMP. The inhibitor (100 µg/ml) was added to the bacterial cells in RPMI medium, just before exposing them to the macrophages and the effect of the treatment was monitored every hour for four hours. As shown in Fig. 3a, AcrB inhibition significantly affects the viability of intracellular bacteria**.** A mild increase of intracellular dead bacteria (10%) in the presence of NMP was observed already at one hour p.i. (T1). This proportion reaches 21% at two hours p.i. (T2) and further rises (25%) at time point T3. At four hours p.i. the percentage of intracellular dead bacteria slightly decreases although it remains significantly higher than in the absence of the NMP inhibitor. Interestingly, the AcrB transporter function clearly influences the behavior of LF82 bacteria facing the host cell environment, as bacterial viability in the extracellular environment is comparable with or without the NMP compound (data not shown). By and large, the data we obtained from AcrB inhibition experiments follow those from experiments performed with the LF82 *ΔacrB* mutant, indicating that the efflux function of AcrB is required for LF82 survival inside the macrophage phagolysosome.

To further determine whether the observed decrease in viability of intracellular bacteria was due to loss of AcrB function, we constructed a LF82 derivative incapable of efflux activity via AcrB. To this end we first generated the p*acrB* plasmid carrying the *acrB* gene (Supplementary Table S1). Then, the A at position 1223 of *acrB* was replaced with C in the plasmid p*acrB* giving rise to the D408A amino acid substitution in the AcrB protein (p*acrB*_D408A_). It has been demonstrated that in *Salmonella typhimurium* the D408A substitution disrupts proton translocation in the pump and abolishes efflux activity^27^. Both p*acrB* and p*acrB*_D408A_ plasmids were used to transform the LF82 *ΔacrB* strain, obtaining the LF82 *ΔacrB* p*acrB* and LF82 *ΔacrB* p*acrB*_D408A_ derivatives, respectively. The latter strain displays a membrane protein composition similar to that of the wt, with the AcrB protein lacking the transporter function. To evaluate if the amino acid substitution could affect bacterial fitness, we analyzed the growth properties of LF82 and LF82 *ΔacrB* pacrB_D408A_ in LB medium. The results show that LF82 *ΔacrB* p*acrB*_D408A_ has a similar generation time as the wt, indicating that the mutation does not confer a detectable growth defect in LB medium (Supplementary Fig. S1). We also determined the susceptibility of the LF82 *ΔacrB* p*acrB*_D408A_ strain to bile salts and antibiotics. As for the LF82 *ΔacrB* mutant, also LF82 *ΔacrB* p*acrB*_D408A_ is hypersusceptible to bile salts, with a twofold dilution decrease in the MIC as compared to the parental strain (Table 1). As expected, the susceptibility of the LF82 *ΔacrB* p*acrB* derivative to bile salts and antibiotics matches that of wt strain.

To assess whether the LF82 *ΔacrB* strain expressing an unfunctional AcrB still showed impaired survival ability, we compared the viability of LF82 wt with that of LF82 *ΔacrB* p*acrB* and LF82 *ΔacrB* p*acrB*_D408A_ derivatives during macrophage infection by determining the percentage of PI positive intracellular bacteria at different time points p.i. As shown in Fig. 3b already at one hour p.i. (T1) cell death caused by the expression of the unfunctional AcrB D408A is almost seven-fold higher (28%) than in the wild type (4%). It further increases (40%) at two hours p.i. (T2) and remains at approximately this value throughout the infection period. Importantly, the viability curve of intracellular LF82 *ΔacrB* p*acrB* overlaps that of the parental strain. The phenotype conferred by the unfunctional AcrB D408A was further corroborated by determining the viable bacteria by the CFU assay. As shown in Fig. 3c, the CFU/ml at representative time points (T0, T2 and T5) nicely mirror the survival curves defined by the “Live and Dead” assay. These results further confirm that the efflux activity associated with AcrB is critical for LF82 persistence and multiplication inside macrophages, ruling out the possibility that the effect observed in the absence of AcrB might be due to the simple lack of this large and abundant inner membrane protein.

## Discussion

We present evidence supporting the crucial role of AcrAB EP in the infection process of a prototype AIEC strain, LF82. In particular, our data, based on infection experiments with mutant strains lacking efflux pump components AcrB and/or AcrA, clearly indicate that both proteins are required for AIEC persistence and replication inside THP-1 derived macrophages. Furthermore, chemical inhibition of AcrB transporter activity in the wt LF82 and loss of function experiments point to a pivotal role of the AcrB efflux function in the survival of AIEC inside the macrophage phagolysosome.

As a major efflux pump in *Escherichia coli*, *Salmonella,* and other Enterobacteriaceae AcrAB-TolC is often used as model of the RND family, the main class of efflux pumps (EPs) involved in the emergence of multidrug resistance (MDR). The physiological role of the AcrAB EP is much more complex than that of a simple antibiotic export system as it is known to confer resistance to a variety of compounds (e.g. dyes, detergents, and host-derived molecules)^1,9^. In particular, in *E. coli* AcrAB contributes to the colonization and adaptation of the bacteria to the intestinal tract by exporting bile salts^8,44^, and its function has been associated with the virulence of several pathogenic bacteria^21,29,30,32,33,45^. Among different *E. coli* pathogens, AIEC represent a specific group associated with Chron’s disease, a severe intestinal inflammation syndrome. AIEC colonize intestinal epithelial cells promoting the disruption of the intestinal barrier^34,39^. They also enter macrophages, persisting and replicating within phagolysosomes without triggering cell death^40^. Persistence inside this harsh environment requires prompt bacterial adaptation, which implies the ability to keep the bacterium free of dangerous compounds. In this respect, we have previously reported how the expression of MDR EPs encoded by AIEC strain LF82 is specifically modulated during the infection process. In particular, we found that the expression levels of *fsr* and *mdtL*, which encode MDR EPs of the MFS family, and *mdtE,* which encodes an EP of the RND family, are highly expressed in macrophages^24^. We were also able to prove that the MdtEF EP is involved in increasing the fitness of AIEC inside macrophages.

In the present study we demonstrate that AcrAB contributes to the survival of AIEC LF82 in the macrophage environment as evidenced by increased bacterial death after infection of THP1-derived macrophages with a AcrAB-defective mutant (LF82 *ΔacrAB)*. The percentage of dead LF82 *ΔacrAB* bacteria is higher than that of the wt strain throughout the infection period considered. The capability of ectopically expressed *acrAB* genes to restore the intracellular viability in LF82 *ΔacrAB* confirms the contribution of the AcrAB EP to the fitness of AIEC in the macrophage niche and highlights the relevance of this EP in such a hostile environment.

It is known that AcrA and AcrB have specific functions and contribute to the full functionality of the EP. The evidence presented here indicates that the inactivation of AcrA or AcrB both leads to decreased bacterial viability in macrophages. These data are in agreement with the results obtained in other pathogenic bacteria showing that each component of the AcrAB complex plays a specific role in the virulence, pathogenesis and survival within infected host cells^26,29,45^. Interestingly, we observed that the percentage of dead LF82 *ΔacrA* cells during macrophage infection is lower than that of the LF82 *ΔacrB* strain. It has been recently reported that the AcrB transporter is able to interact with the periplasmic adapter protein AcrE^46^, which might functionally compensate the absence of AcrA thus explaining the milder death percentage of LF82 *ΔacrA* and highlighting the predominant role of AcrB in the extrusion of toxic compounds present in the phagolysosome. The relevance of AcrB is further strengthened by the inactivation of the EP achieved by adding a specific inhibitor (NMP) during infection of macrophages. The presence of NMP impairs the LF82 viability at very early time point of infection (Fig. 3a). We were also able to rule out that the inability of LF82 *ΔacrAB* and LF82 *ΔacrB* to survive inside macrophages is due to a profound alteration of the inner membrane because of the lack of an abundant constituent such as AcrB. Indeed, complementation of LF82 *ΔacrB* with an AcrB protein (AcrB D408A) lacking the efflux activity does not recover the wild-type viability (Fig. 3b,c).

Overall, our data support the view that in order to better survive intracellularly LF82 cells require the ability of AcrB to select and bind different substrates, thus allowing them to escape the dangerous compounds present in the inhospitable macrophage environment. It has been reported that in *Salmonella* the AcrAB EP is involved in the infection of both macrophages and epithelial cells, being required for adhesion to and invasiveness of host cells^21,26,27^. Here we mainly focused on the requirement of the AcrAB EP for LF82 survival inside host cells, demonstrating that its activity is needed for AIEC persistence and replication within the human THP-1-derived macrophages, allowing a successful infection. As for the role of AcrAB during the infection of epithelial cells we find that it does not influence the survival of LF82 within Caco-2 cells. The evidence we have presented here confirms the clear involvement of AcrAB in the interactions between host cell and bacteria and extends the range of bacterial pathogens that exploit the AcrAB function to cope with the hurdles encountered in the intracellular environment.

## Methods

### Bacterial strains, plasmids and growth conditions

Bacterial strains and plasmids used in this study are listed in Supplementary Table S1. *Escherichia coli* LF82 is an AIEC strain isolated from a chronic ileal lesion of a Chron Disease patient^34^. *E. coli* DH10b has been used as the recipient in cloning experiments. Strains LF82 *ΔacrAB*, LF82 *ΔacrB*, and LF82 *ΔacrA* have been constructed using the one-step method of gene inactivation^41^ by transforming LF82 pKD46 with an amplicon obtained using pKD13 or pKD4 as template and the oligo pairs ABF/ABR for the *acrAB* deletion, BF-BR for the a*crB* deletion and AF-AR for the *acrA* deletion (Supplementary Tables S1 and S2). Plasmids p*acrAB* and p*acrB* were obtained by cloning the *acrAB* and *acrB* genes respectively into pGIP7, a pACYC184-derived vector carrying the ptac promoter^47^ (Supplementary Table S1). The *acrAB* and *acrB* amplicons obtained using LF82 as template and oligo pairs pAC*acrAB*F/pAC*acrAB*R and pAC*acrB*F/pAC*acrB*R respectively (Supplementary Table S2) were digested with BamHI and cloned into pGIP7 downstream the ptac promoter. Plasmids p*acrAB* and p*acrB* and the size of the of *acrAB*, *acrB* and *acrA* deletions have been verified by DNA sequencing (Biofab, Rome). The D408A mutation of *acrB* was obtained by a Site-Directed Mutagenesis System (GENEART^®^) using the oligo pair pAC*acrB*-D408A F/pAC*acrB*-D408A R (Supplementary Table S2). In particular, we replaced the A at position 1223 (from the ATG of acrB) with C, generating a codon change from GAC to GCC (D408A). The presence of the correct mutation on p*acrB*_D408A_ has been verified by DNA sequencing (Biofab, Rome).

Growth kinetics of LF82 and its derivatives in different media (LB, DMEM or RPMI) were measured using a CLARIOstar plate reader (BMG LABTECH, Offenburg, Germany). When required, Congo Red was added (0.01%) to Trypticase soy agar to monitor the Congo Red phenotype (CR+). Antibiotics and inhibitors were used at the following concentrations: ampicillin 30 µg/ml, chloramphenicol 25 μg/ml, ciprofloxacin 0.0015 to 0.25 μg/ml; erythromycin 25 to 200 μg/ml; kanamycin 30 μg/ml; gentamicin 10 μg/ml or 100 μg/ml for infection procedures; tetracycline 0.25 to 1 μg/ml; 1-(1-naphthyl-methyl)-piperazine (NMP) 100 μg/ml. Bile salts were used at concentrations ranging from 750 to 1500 μg/ml when required.

### General procedures

DNA purification, restriction, cloning, plasmid transformation and gel electrophoresis were perfomed as described^23,47^. Oligonucleotides were based on the LF82 genome^48^ and are listed in Supplementary Table S2. PCR reactions were set up with DreamTaq DNA polymerase (Thermo Fisher Scientific) or Ex taq DNA polymerase (Takara). DNA and protein sequence comparisons were performed using the BLAST Server (National Center for Biotechnology Information).

### Determination of MICs of different compounds

MICs were determined following previously established protocols as recommended by the European Committee on Antimicrobial Susceptibility Testing (EUCAST) (EUCAST 2000). In particular, strains LF82, LF82 *ΔacrAB*, LF82 *ΔacrA,* LF82 *ΔacrB* and LF82 *ΔacrAB* p*acrAB*, LF82 *ΔacrB* p*acrB* and LF82 *ΔacrB* p*acrB*_D408A_ were inoculated into 3 ml of LB and grown at 37 °C with shaking for 16 h. Cultures were then diluted to OD600 0.02 in LB and 100 µl aliquots were transferred to microtiter wells containing 100 µl of the compounds to be tested (at appropriate concentrations). After 16 h incubation at 37 °C growth was estimated by measuring the OD600 of each well. At least two biological replicates were performed.

### Cell cultures and infections

The THP-1 cell line was grown in RPMI 1640 (Gibco) medium containing 10% heat-inactivated fetal bovine serum (FBS) (Euroclone), 0.05 IU/ml penicillin and 0.05 IU/ml streptomycin (PS), referred to as RF10, at 37 °C in a humidified 5% CO_2_ atmosphere. Before bacterial infection, THP-1 monocytes were differentiated into macrophages. Cells were seeded in 6-well tissue culture plates (Falcon) at 1.0 × 10^6^ cells/well in RF10 supplemented with 50 nM PMA. After 48 h, PMA containing medium was removed and cells left for further 24 h in RF10. Two hours before bacterial addition, RF10 was replaced with fresh RPMI without serum and antibiotics. The human epithelial colorectal adenocarcinoma Caco-2 cell line was grown in Dulbecco minimal essential medium (DMEM) (Gibco) containing 10% FBS and PS, referred to as DF10, at 37 °C in a humidified 5% CO_2_ atmosphere. For bacterial infection cells were seeded in 6-well tissue culture plates (Falcon) at 4.0 × 10^5^ cells/well in growth medium. After 48 h cells were serum-starved over-night in DMEM supplemented with 0.5% FBS and PS (DF0.5). Two hours before bacterial infection, the medium was replaced with fresh DMEM without serum and antibiotics. Bacteria were added to the cell cultures at MOI 100. The plates were then centrifuged (15 min, 750×*g*) and incubated 30 min (THP-1) or 45 min (Caco-2) at 37 °C under 5% CO_2_ atmosphere to allow bacteria to enter the cells. Finally, extracellular bacteria were removed by washing three times with PBS. This point was taken as time zero (T0). Fresh medium (RPMI or DMEM) containing gentamicin (100 μg/ml) was added to kill extracellular bacteria, and infected cells were incubated at 37 °C up to 4 or 5 h.

### Live and dead assay and viable bacterial count

Intracellular dead bacteria were evaluated by staining the entire population with DAPI (Sigma) and labelling dead cells with Propidium Iodide (PI) (Sigma). At the indicated time points, infected cells were lysed by adding 1% Triton X-100 in 1 × PBS. The cell lysate was pelleted at 13,000 rpm for 5 min. The pellet containing intracellular bacteria was washed once in 1 × PBS and suspended in 1 × PBS containing 10 μg/ml DAPI and 15 μM PI. Samples were incubated 20 min at room temperature in the dark. Bacteria were centrifuged at 13,000 rpm for 5 min and washed once with 1 × PBS. Pellet was resuspended in 20 μl 1× PBS containing 50% glycerol, 5 μl of the stained bacteria were added to the glass slide and overlaid with the coverslip for immediate observation. Stained cells were examined using the NIKON ECLIPSE 50i fluorescence microscope. Single images were recorded on QICAM FAST 1394 camera (Qimaging) and imported to ImageJ software for processing (Supplementary Fig. S2).

To define the number of viable intracellular bacteria, macrophage infection was carried out as describe above. At the indicated time points, after lysing the host cells with 1% Triton X-100, intracellular bacteria were collected, washed and resuspended in PBS. Serial dilutions of the bacterial suspensions were plated on LB agar plates to calculate the CFU/ml.

### LDH cytotoxicity assay

To verify that the NMP inhibitor does not affect cell viability, cytotoxicity was measured by using CyQUANT™ LDH Cytotoxicity Assay Kit (Invitrogen). THP-1-derived macrophages were grown in RPMI with or without NMP (100 µg/ml) in tissue culture dish (35 X 10 mm, Falcon) at 1.0 × 10^6^ cells/well in growth medium and LDH activity in the medium was determined after 2- or 4-h treatment following manufacturer instructions. Samples containing maximum LDH activity and spontaneous LDH activity were also prepared as well as the LDH positive control. The absorbance has been measured at 490 nm and 680 nm with a CLARIOstar plate reader (BMG LABTECH, Offenburg, Germany) and the percentage of cytotoxicity was calculated according to the manufacturer’s instructions.

### Statistical analyses

The statistical difference between the percentage of dead intracellular bacteria of the LF82 wt strain and its derivative strains at each time point was determined by a two-tailed student’s t-test. The experiments were run at least in triplicate.

## Supplementary Information

Supplementary Information.

## Data Availability

The datasets used and/or analysed during the current study are available from the corresponding author on reasonable request.
