# Supplementary material for: AcrAB efflux pump impacts on the survival of adherent-invasive Escherichia coli strain LF82 inside macrophages
Source: Sci Rep. 2023 Feb 15;13:2692. doi: 10.1038/s41598-023-29817-0 (PMC9931695; doi:10.1038/s41598-023-29817-0)
Supplement: Supplementary file 1 — Supplementary Information. [file 41598_2023_29817_MOESM1_ESM.pdf]

Supplementary Materials

Table S1 Strains and plasmid used in this study

Strains	Characteristic	Source/References
DH10b	$[mcrA\ (mrr - hsdRM\ - mcrBC)\ lacX74\ deoR\ recA1]$	*Sambrok and Russel, 2001*
LF82	AIEC clinical strain isolated from ileum biopsy of a CD patient used as prototype	*Boudeau et al.*, 1999
LF82 *ΔacrAB*	LF82 derivative defective in *acrAB* genes, Km^R	This study
LF82 *ΔacrB*	LF82 derivative defective in *acrB* gene, Km^R	This study
LF82 *ΔacrA*	LF82 derivate defective in *acrA* gene, Km^R	This study
**Plasmids**		
pKD46	Temperature sensitive replicon that carried bacteriophage λ red genes (γ, β and *exo*) under control of arabinose inducible P*araBAD* promoter, Ap^R	*Datsenko and Wanner, 2000*
pKD13	Kan^R containing plasmid, template for PCR	*Datsenko and Wanner, 2000*
pKD4	π-dependent plasmid carrying kanamycin resistance gene flanked by the recognition sites of the yeast FLP recombinase in direct repeats	*Datsenko and Wanner, 2000*
pGIP7	pACYC184-derived vector carrying *lacI-lac* promoter region, Cm^R	*Falconi et al., 2001*
p*acrAB*	pGIP7 derivative plasmid carrying the *acrAB* gene, Cm^R	This study
p*acrB*	pGIP7 derivative plasmid carrying the *acrB* gene, Cm^R	This study
p*acrB* D408A	p*GIP7* derivative plasmid carrying the *acrB* $_{D408A}$ allele, Cm^R	This study

Table S2 Oligonucleotides used in this study

Chromosomal mutations	
**Name**	**5'-3' sequences**
ABF	ACTTTTGACCATTGACCAATTTGAAATCGGACACTCGAGGTTTACATATGTGTAGGCTGGAGCTGCTTCG
ABR	TAGGCATGTCTTAACGGCTCCTGTTTAAGTTAAGACTTGGACTGTTCAGGATTCCGGGGATCCGTCGACC
BF	TATAAAAAAGGCCGCTTGCGCGGCCTTAGTGATTACACGTTGTATCAATGATTCCGGGGATCCGTCGACC
BR	CCTGAACAGTCCAAGTCTTAACTTAAACAGGAGCCGTTAAGACATGCCTATGTAGGCTGGAGCTGCTTCG
AF	ACTTTTGACCATTGACCAATTTGAAATCGGACACTCGAGGTTTACATATGTGTAGGCTGGAGCTGCTTCG
AR	TAGGCATGTCTTAACGGCTCCTGTTTAAGTTAAGACTTGGACTGTTCAGGCATATGAATATCCTCCTTA

Construction of plasmids	
**Name**	**5'-3' sequences**
pAC*acrAB*F	NNNGGATCCATGACCAATTTGAAATC
pAC*acrAB*R	NNNGGATCCTGAGTTGGTGGTTCAATACT
pAC*acrB*F	NNNGGATTCATGCCTAATTTCTTTATCGATCGC
pAC*acrB*R	NNNGGATTCGGATCCTGAGTTGGTGGTTCAATACT
pAC*acrB* $_{D408A}$ F	CATCGGCCTGTTGGTGGATGCCGCTATCGTTGTGGTAGAAA
pAC*acrB* $_{D408A}$ R	TTTCTACCACAACGATAGCGGCATCCACCAACAGGCCGATGG

Fig. S1

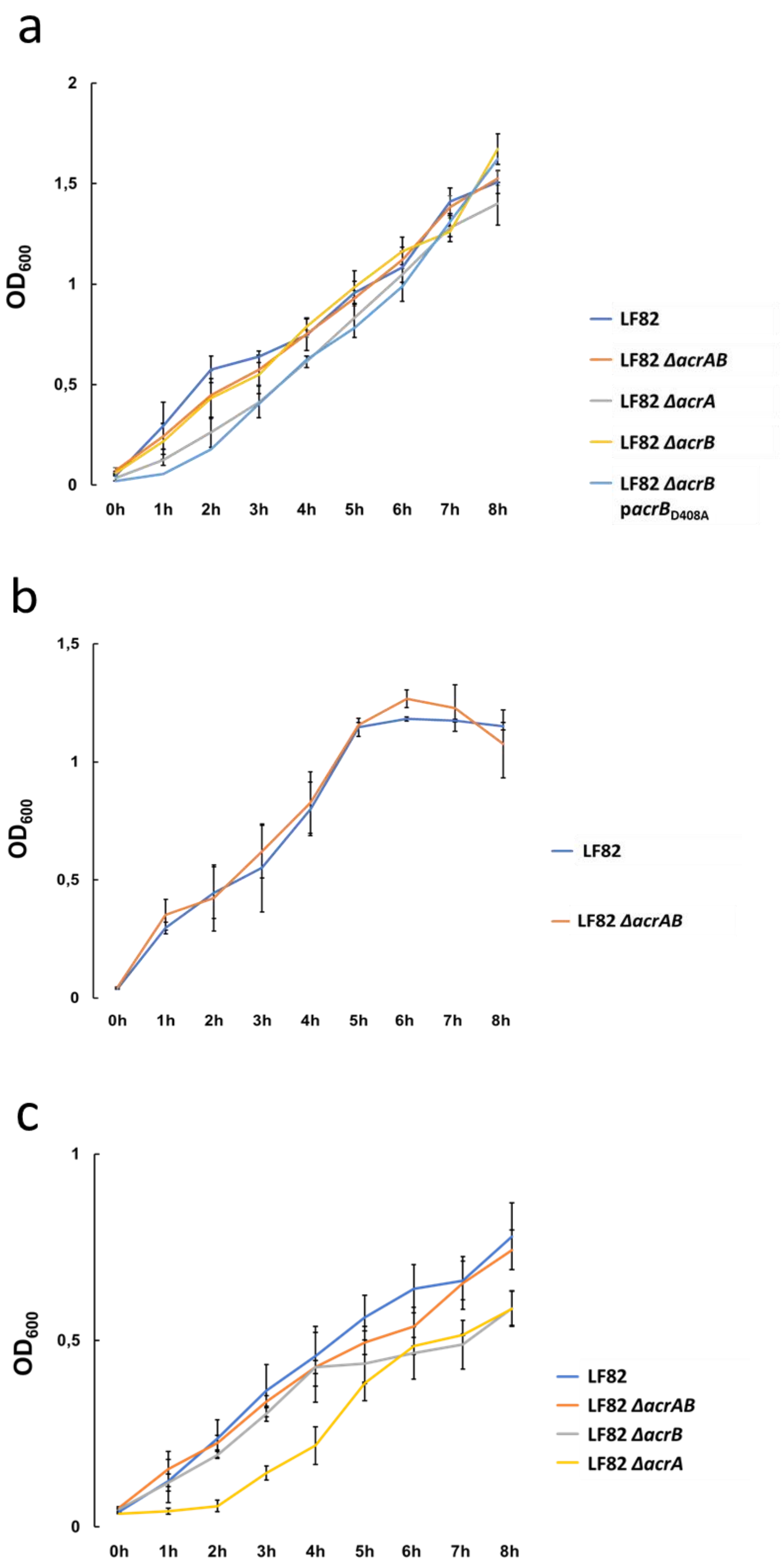

Figure S1. Growth of LF82 and its derivatives. AIEC LF82 and the indicated derivatives were grown in LB medium **(a),** DMEM **(b)** and RPMI **(c)**. The results are the average of at least three independent experiments. Error bars represent the SD.

Fig. S2

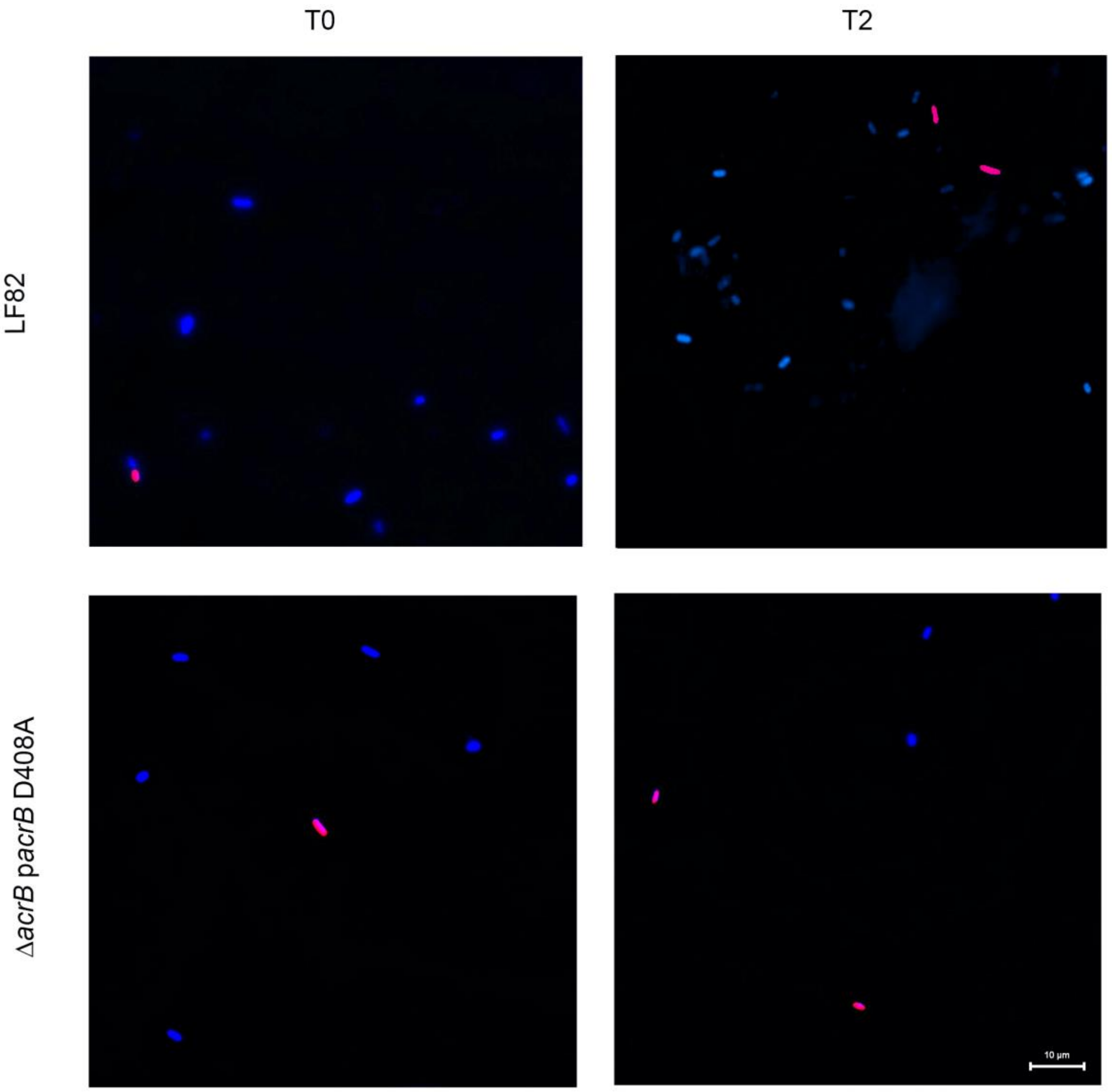

Figure S2. Live and dead assay. Images of DAPI (blue)/PI (red) double stained intracellular LF82 and LF82 Δ*acrB* p*acrB*$_{D408A}$ bacteria recovered at the indicated time points. The pink colored bacteria result from the overlapping of the red (PI) and blue (DAPI) colors and represent dead bacteria.
